# Auscultatory Sign of Enlarged Liver

**Published:** 1850-12

**Authors:** 


*Auscultatory Sign of Enlarged Liver.*—Br. Walshe has described in the *Lancet*, a stethoscopic indication of enlarged liver, under the name of "*hepatic compression rhonchus.*"

"It coexists with inspiration only, or indeed, seems to be rather superadded to it, not commencing until the inspiration-murmur appears almost at an end. Its evolution is peculiarly slow, drawling, and (if I may be allowed the expression) lazy, being, in this respect the exact reverse of that of the crepitant rhonchus of pneumonia. It consists of a variable (but commonly a great) number of excessively fine, dry crepitations, rather superficial than deep-seated; is rendered audible by forced inspiration only, and may be heard in front, at the side, and in the back of the right half of the chest (least commonly in front, however,) at or near to, the upper edge of the liver. Its existence is completely independent of any lung-affection; and I have never found it on the left side, in these cases of liver-enlargement. The characters of this rhonchal sound are so peculiar, that a mere tyro would be able to distinguish it from all varieties of rhonchus,—it differs essentially, as I have just proved, from crepitant, subcrepitant, and dry crackling, pulmonary rhonchi, and from pleural rhonchus. Of its mechanism I am not prepared, at present, to offer any demonstration; but it appears to me to be most feasibly explicable as follows;—The lower portions of the lung, pressed upon by the enlarged liver, undergo a sort of creasing, or condensation, which, in ordinary breathing, interferes with their expansion. By forced inspiration, the portion of lung implicated will readily be understood to be *uncreased*, and so conceivably a series of sounds, such as I have described, is produced. Another fact strongly corroborates this hypothesis—namely, that it often ceases to be audible, for a time, after from one to five or six forced inspirations: the lung seems to require rest and time to be again creased up. Should further experience confirm this view of its mode of production, we shall have collateral support given to the doctrine I have long taught (and which, so far as I know, has not been refuted,) that the crepitant rhonchus of pneumonia is formed, not in the air-cells or capillary bronchi, but in the pulmonary parenchyma itself. I am not able, as yet, to make any positive assertion concerning the frequency with which the rhonchus under consideration attends enlargement of the liver; but, on the other hand, I am in a position to affirm, that in no single case of notable increase of bulk of that organ which has fallen under my observation, since my first discovery of the rhonchus, have I failed to substantiate its existence. The sound may, it is true, escape detection on one or more occasions, but has never been absent for a series of days. On the other hand, I have not met with it in other conditions of disease; though doubtless, if my theory concerning its formation be well founded, it will probably be ascertained to accompany a variety of conditions, causing slight compression of the lung."—*London Journal of Medicine.*

